# A new cell line (8701-BC) from primary ductal infiltrating carcinoma of human breast.

**DOI:** 10.1038/bjc.1989.248

**Published:** 1989-08

**Authors:** S. Minafra, V. Morello, F. Glorioso, A. M. La Fiura, R. M. Tomasino, S. Feo, D. McIntosh, D. E. Woolley

**Affiliations:** Istituto di Istologia ed Embriologia, UniversitÃ di Palermo, Italy.

## Abstract

**Images:**


*Br. J. Cancer* (1989), **60**, 185–192 © The Macmillan Press Ltd., 1989

### A new cell line (8701–BC) from primary ductal infiltrating carcinoma of human breast

S. Minafra[1], V. Morello[2], F. Glorioso[1], A.M. La Fiura[1], R.M. Tomasino[2], S. Feo[3], D. McIntosh[4] & D.E. Woolley[4]

[1]*Istituto di Istologia ed Embriologia;* [2]*Cattedra di Istologia ed Anatomia Patologica R;* [3]*Istituto di biologia dello sviluppo (CNR), Università di Palermo, Via Archirafi 20–22, 90123 Palermo, Italy;* [4]*University Hospital of South Manchester, West Didsbury, Manchester M20 8LR, UK.*

**Summary** A cell line, designated 8701–BC, was established in culture from tissue fragments of primary ductal infiltrating carcinoma of human breast. The cell cultures after the sixth passage were devoid of contaminating fibroblasts as judged by the positive staining of all cells with the specific epithelial cell markers carcinoembryonic antigen (CEA), tissue polypeptide antigen (TPA) and cytokeratin 8. The epithelial nature of these cells was confirmed by ultrastructural analyses which demonstrated the retention of specific structural properties characteristic of the original tumour. The cells possessed an abnormal karyotype with 55–60 chromosomes per cell with numerous rearrangements. They do not express HLA antigens and the c-*myc* gene was not amplified. The 8701–BC cells have a doubling time of approx. 29h and have been maintained in culture for more than 100 passages. These properties suggest the establishment of a human neoplastic cell line which, with its ability to produce homotrimer collagen *in vitro*, will provide a useful model system for the study of tumour cell:stromal matrix interactions.

Previous ultrastructural and biochemical studies on ductal infiltrating carcinomas (d.i.c.) of human breast have suggested that interactions between the tumour cells and extracellular collagenous matrix are important for the invasive behaviour of these neoplastic cells (Minafra *et al.*, 1984*a,b*, 1988; Pucci Minafra *et al.*, 1985, 1986, 1987). To examine this further we have attempted to establish a breast carcinoma cell line *in vitro* which retains many of the properties observed *in vivo*. To date the majority of breast cancer-derived cell lines have been obtained from secondary tumours and pleural effusions (Dobrynin, 1963; Soule *et al.*, 1973; Trempe & Fogh, 1973; Engel & Yung, 1978; Monaghan *et al.*, 1985). Although a cell line derived from a primary breast carcinoma has recently been reported (Vandewalle *et al.*, 1987) the relative paucity of cell lines derived from primary carcinomas (Hackett *et al.*, 1977; Nordquist *et al.*, 1975; Lasfargues *et al.*, 1978; Rudland *et al.*, 1985) may be related to the technical difficulties associated with the extraction of viable tumour cells from surrounding stroma. Here we report the isolation, establishment and characterisation of a continuous line of neoplastic cells isolated from a primary duct infiltrating carcinoma of human breast (8701–BC). These cells have been grown in monolayer cultures for more than 100 passages and have retained morphological characteristics similar to those of the original tumour.

### Materials and methods

### *Tissue fractionation*

Tumour fragments obtained from surgical operations and histologically diagnosed as ductal infiltrating carcinoma were washed under sterile conditions with a $Ca^{2+}$ and $Mg^{2+}$ – free balanced salt solution (BSS-CMF). Specimens to be used for cell culture were taken from the core of the tumour, cut into very small pieces and pre-incubated in BSS-CMF with gentle stirring at 37°C for 15 min. The tissue was then transferred to BSS-CMF containing 0.125% (w/v) collagenase (Sigma type III), 0.1% (w/v) Hyaluronidase (Sigma) and 0.4% (w/v) demineralised bovine serum albumin (BSA) (Calbiochem grade), and digested for 45 min at 37°C with gentle rotation. The resulting cell suspension was centrifuged for 2 min at 300*g* and the pelleted cells were resuspended in 5ml culture medium supplemented with 20% (w/v) fetal calf serum (FCS) and 10% (v/v) Tryptose Phosphate Broth (TPB) (Difco).

8701–BC cell line was derived from a G2–G3 duct infiltrating carcinoma obtained from a 72-year-old patient, with extensive lymphonodal infiltration. Histological grading was evaluated according to Bloom & Richardson (1957).

### *Cell culture*

Cells of primary cultures were grown in minimum essential medium (MEM) with Earle's salts (Biochrom) supplemented with 20% (v/v) FCS (Difco) and 10% (v/v) TPB (Difco). A cell density of $1–2 \times 10^5$ cells $ml^{-1}$ was used to seed primary cultures in 5ml flasks. At confluency cells were subcultured following detachment by exposure to trypsin 0.5% (w/v) and Versene 0.04% (w/v) in $Ca^{2+}$, $Mg^{2+}$-free phosphate buffered saline (PBS) for 5 min. Cells were resuspended in fresh medium and subcultured in RPMI 1640 medium (Gibco) with 10% FCS and 5% TPB in flasks or dishes, according to experimental needs.

### *Electron microscopy*

Seven days after seeding the cells were fixed *in situ* for 90 min at 4°C with 2% (w/v) paraformaldehyde/2.5% (w/v) glutaraldehyde in 0.1M sodium cacodylate buffer at pH 7.4 containing 8 mM $CaCl_2$. After several washings the cells were post-fixed with 2% (w/v) $OsO_4$ in the same buffer for 1h, washed again and gently scraped from the bottom of the flask. The cells were collected by low speed centrifugation, dehydrated with a graded series of alcohols and embedded in Araldite. Ultrathin sections were stained with 1% (w/v) phosphotungstic acid followed by 1% (w/v) uranyl acetate and examined using a Philips EM420 electron microscope.

### *Immunocytochemistry*

For immunocytochemical staining subcultured cells were plated at different densities on glass coverslips in dishes and incubated for at least 24h. The cells were fixed for 30 min in cold acetone and rehydrated through several washes in PBS. Tissue polypeptide antigen (TPA), carcinoembryonic antigen (CEA) (Bjorklund *et al.*, 1982) and cytokeratin 8 ('Cytokeratin A', Moll *et al.*, 1982) were used as epithelial cell markers.

Correspondence: S. Minafra.
Received 20 December 1988, and in revised form, 20 March 1989.

An antibody against HLA antigen was also tested as a human cell marker. Serum anti-TPA (Byk-Guldon), anti-CEA (Ortho-diagnostic) and anti-HLA-A-B-C (Technogenetics) were used as purchased; serum anti-cytokeratin 8 (Orthodiagnostic) was diluted 1:250 with PBS pH 7.6 containing bovine albumin.

The peroxidase-anti-peroxidase (PAP) method (Sternberger, 1979) was used to detect TPA, cytokeratin and HLA antigens with slight modifications of the Ortho-diagnostic kit. For CEA reactions the avidin-biotin method was used according to Hsu *et al.* (1981). Substrate for the PAP-reaction was 3-amino-9-ethylcarbazole (AEC) and for CEA 3-3′-diaminobenzidine (DAB) was used. All reactions were carried out in a humidified incubator at room temperature except for the incubation with the primary antiserum which was at 4°C overnight. After immunolocalisation the samples were counterstained with Mayer's haematoxylin solution. Colonic adenocarcinomas were used as positive controls for CEA, TPA and cytokeratin 8 determinations and human lymphocytes were used for HLA positive controls. For negative controls the cell cultures were treated with non-immune serum in place of the primary antiserum, and normal human breast epithelial cells were tested for CEA antigen.

*c-*myc *oncogene restriction pattern and expression*

DNA from 8701-BC, DAUDI (human lymphoma cells) and PAF (human fibroblast cells) (Dalla Favera *et al.*, 1983) were subjected to Southern transfer analysis. DNA samples were digested with EcoRI restriction enzyme and fractionated (10$\mu$g of DNA per lane) by 0.8% (w/v) agarose gel electrophoresis in 40mM tris-HCl containing 5mM NaOAc, 2mM EDTA, pH 8.0. Hind III digested $\lambda$-phage DNA were included in gels as molecular weight markers. Transfer of DNA from the gel to nitrocellulose sheet was performed as described by Southern (1975). The nitrocellulose sheet was probed with $^{32}$P-labelled human c-*myc* (Ryc 7.4) as described by Feo *et al.*, (1986). For c-*myc* expression, Northern blot analysis was carried out. Cellular cytoplasmic RNAs were isolated from 8701-BC cells, DAUDI, PAF and HL60 (human promyelocitic cells) (Collins *et al.*, 1977) according to the method of Berger & Birkenmeier (1979). The RNAs were denatured in 6% (w/v) formaldehyde: 50% (v/v) formamide at 50°C for 15 min and subjected to electrophoresis in 1% (w/v) agarose gel containing formaldehyde (10$\mu$g of RNA per lane). The fractionated RNAs were electroblotted on to Nytran membranes and hybridised with the c -*myc* probe as described by Feo *et al.*, (1986).

*Preparation of metaphase chromosome spreads*

Cells were plated out at subconfluency in 25cm$^2$ tissue culture flasks overnight. On the following day, 150$\mu$l of *N*-deacetyl-*N*-methyl colchicine (stock solution 100$\mu$g ml$^{-1}$) was added to each 5ml culture and the flasks were incubated at 37°C for 3 h. The cells were detached from the culture vessel by trypsinisation and pelleted by centrifugation at 1000 rpm for 5 min in culture medium containing 5% (w/v) FCS. The supernatants were removed, leaving 250$\mu$l above the cell pellet and a hypotonic solution (0.075 M KCl) was added to a total volume of 4ml. The pellet was gently dispersed and the cells were incubated at room temperature for 6 min. The cells were then centrifuged at 750 rpm for 6 min, the supernatant was removed and the cells were then fixed three times in freshly prepared ice cold fixative (3:1 ethanol/glacial acetic acid). The cells were resuspended in 500$\mu$l of fresh fixative and two or three drops of the suspension were dropped on to clean, chilled, wet glass slides. The preparation was dispersed by gently blowing on the slides which were then left to dry at room temperature. The slides were stained in 10% Giemsa pH 6.8 for 10 min and rinsed in tap water.

*Trypsin-Giemsa banding of chromosomes*

Unstained preparations were treated with $H_2O_2$ (20 vol) for 5 min at room temperature and slides were then immersed in a 0.1% (w/v) trypsin/PBS solution ($Ca^{2+}$ $Mg^{2+}$ free) for 30 s to 2 min. The cells were then stained in Giemsa and rinsed in tap water. The chromosomes were viewed under the microscope to assess the degree of banding. Insufficiently banded preparations were destained using fixative, washed in PBS and retrypsinised. Satisfactory preparations were mounted using Histomount and viewed at high power on a Zeiss Photomicroscope III.

*Karyotypic analysis*

Chromosome counts were carried out on 50 unbanded preparations to obtain a mean chromosome number. The trypsin-Giemsa-banded preparations were examined in order to identify individual chromosomes and in the construction of a karyotype. Individual metaphase spreads were photographed on a Zeiss Photomicroscope III using Kodak panatomic-X film.

### Results

*Cell cultures*

The primary cell cultures grew slowly, taking 2–3 weeks to become confluent. Mesenchymal cells predominated over

**Figure 1 a,** Primary cell culture from d.i.c. tissue fragments. After 3 weeks of incubation the majority of cells appeared as long, interlacing, spindle-shaped elements with scattered colonies of a different cell population. × 81. Inset: higher magnification of a selected area of the preceding field showing a nest of a different cell population growing on underlying mesenchymal cells. × 162. **b,** Cell culture after five first passages and 2 weeks of incubation. The aggregate formation on a layer of mesenchymal cells was more evident in secondary cultures in which the number of neoplastic cells was proportionally higher. Plating cell density (pD) < 3.5 × 10$^4$cm$^{-2}$. × 162.

epithelial cells and appeared at this stage as long interlacing, spindle-shaped elements (Figure 1a). Nevertheless, nests of a different cell population (the presumed neoplastic cells) were present as scattered colonies growing on the underlying film of mesenchymal cells (Figure 1a, inset). These colonies became progressively larger when allowed to age a few days after confluence and tended to unite to form large multi-layered aggregates (Figure 1b). In subsequent passages, due to different proliferation rates, the colony-forming cells overcame the mesenchymal ones, and from the sixth passage they were the predominant cell population in all cultures.

The nature of the colony-forming cells was examined using assays for three specific epithelial markers at different stages of subculture. The tests carried out on hundreds of samples showed that all the cells were positive for these epithelial markers and demonstrated that mesenchymal cells were absent from our long-term cultures.

*Cell morphology*

Cell shape and cytoplasmic inclusions showed some variability among the neoplastic population. Twenty-four to 48 h after seeding, all cells had a rather epithelioid shape with large nuclei and often two prominent nucleoli (Figure 2a). Three to four days after seeding they reached confluence and tended to build duct-like hollow structures lined by elongated cells (Figure 2b); later on, smaller polygonal cells of equal size penetrated the hollow structures. After 7 days small translucent cells appeared to invade the upper surface of the culture. If the serum content of the culture medium was lowered to 5% or less, it was possible to keep the culture at this stage for as long as 1 month.

When cells were plated at a density greater than $2.6 \times 10^4 cm^{-2}$, or were kept in culture for about 2 weeks from a lower density, they tended to form colonies growing on the film of confluent elongated cells. These colonies were composed of either darker, elongated cells, or of smaller, rounded, translucent cells in the same flask (Figure 3a). A common feature shown by these cells was their ability to connect with each other by very long cytoplasmic bridges or spikes, sometimes between opposite edges of the hollow structures (Figure 3b, arrow).

**Figure 2 a,** Cell culture at a late passage (36th) and 48 h after seeding. Before confluence all the cells appeared as elongated epithelioid elements with large nuclei and two or more nucleoli. pD$4.6 \times 10^4 cm^{-2}$. $\times$ 162. **b,** Cell culture at a late passage (41st) and 7 days after seeding. Hollow structures lined by elongated cells are observed containing polygonal cells. pD$2.5 \times 10^4 cm^{-2}$. $\times$ 162.

**Figure 3 a,** Colony formation by darker cells supported by elongated cells arranged in layers. Late passage (35th) at pD of $3.4 \times 10^4 cm^{-2}$. $\times$ 81. **b,** Cell culture at an early passage (8th) 15 days after seeding, but recultured after one year of frozen storage. The edges of a hollow structure are crossed by cytoplasmic spikes (arrow). Colonies developed outside the hollows and often showed small, translucent cells. pD$5 \times 10^4 cm^{-2}$.

*Electron microscopy*

The EM analyses of the long-term culture at 7 days after seeding revealed several ultrastructural analogies with the neoplastic cell population observed in tissue specimens of the original human breast d.i.c. where cells of contrasting electron densities were noted. Figure 4a shows an intercellular canaliculum where a large number of microvilli project into the lumen and Figure 4b shows an interdigitated cell contact with poorly differentiated junctional complex. In Figure 4c an intracellular crypt filled with microvillous projections, typical of breast cancer is observed. These ultrastructural characteristics of the cultured cells indicate their epithelial nature as well as their neoplastic origin.

*Cell growth*

The growth rate of the cells was assessed in later passages. Cells were plated at low density ($3 \times 10^3 cm^{-2}$) in 35mm

**Figure 4 a,** Electron micrograph of an ultrathin section of cells at 43rd passage fixed *in situ*, showing a typical intercellular canaliculum lined by microvilli. The arrow indicates an intercellular junction. × 40,000. **b,** 8701-BC at 45th passage: detail of two cells showing adhesion through interlacing microvillous projections. The arrows point to a poorly differentiated junctional complex. × 15,000. **c,** Detail of an intracellular crypt, completely filled by microvillous projections. × 18,000.

Falcon dishes and the culture medium was changed every 3 days. At selected times dishes in triplicate were trypsinised and the cells were counted using a haemocytometer. Under these conditions cells reach confluence 4 days after seeding; but, as is shown by the growth curve in Figure 5, they do not attain the steady state, but continue an exponential growth with a calculated doubling time of 28.8 h.

*Immunocytochemistry*

Figure 6 a and b shows typical immune reactions of late passage cells (34th to 49th) for TPA and cytokeratin-8 antigens. The staining appears as a diffuse network for the entire cell population. A similar pattern was observed for both antigens which was expected due to the homology between TPA and cytokeratins (Weber *et al.*, 1984) Figure 6c shows the immunolocalization of CEA in cell cultures of similar passage. All cells appeared positive for CEA, although not all of them show the same stain intensity. Positive staining of tumour cells was also obtained in tissue sections (not shown). In contrast, fibroblasts present in early cultures and cells obtained from normal human mammary epithelium (using the method of Easty *et al.*, (1980)), were both CEA negative. Whereas the tumour cells reacted positively for TPA, cytokeratin-8 and CEA, when tested for HLA antigen a negative response was shown by all cells.

*c-*myc *oncogene restriction patterns and expression*

Southern blotting analysis of the c-*myc* gene in 8701-BC cells, when compared with those of two human diploid cell lines, showed that the c-*myc* gene is in a normal germline configuration (12.8 kb EcoRI band) and is not amplified (Figure 7a, lane 2). The same EcoRI band was obtained with

**Figure 5** Cell culture at late passages (35th–40th). Cells were plated in 35mm Falcon dishes at a plating density of $3 \times 10^3 cm^{-2}$ in RPMI 1640 growth medium, supplemented with 10% FCS and 5% TPB. Growth medium was renewed every 3 days. Cell counts were performed on the days indicated. Each point represents the mean of three different determinations made in triplicate and is plotted with standard error bars.

two different human cell lines, PAF and DAUDI (Figure 7a, lanes 1 and 3, respectively). Northern blot analysis on cytoplasmic RNA from 8701-BC cells failed to detect any enhancement of transcription by c-*myc* gene (Figure 7b, lane 2). A Burkitt lymphoma cell line, DAUDI, which shows a slightly higher than normal level of expression (Figure 7b, lane 4), and the HL60 cell line, which has an amplified c-*myc* gene and a 10-fold enhanced expression (Figure 7b, lane 3) were used as controls.

### *Chromosome analysis*

Examination of 50 metaphase spreads of cells from the 11th passage revealed a human karyotype with many chromosomal alterations. The number of chromosomes varied between individual metaphases but all had counts of between 55 and 60. Trypsin-Giemsa banding of the preparations showed that only a few normal chromosomes were present and that each metaphase had two marker chromosomes which possessed non-staining regions. The chromosomes positively identified are shown in Figure 8.

### Discussion

This paper describes a new cell line derived from a primary 'scirrhous' human mammary carcinoma. The methods used have been extended to seven different d.i.c. with the same general results, but the observations and data reported here are derived from 8701-BC, which was chosen for long-term culture. The isolation and maintenance of this tumour cell

**Figure 6 a,** Positive immunoreaction with TPA antigen at late passages (34th–39th). The staining appears as a diffuse network throughout the cytoplasm of the entire cell population. Cell culture on coverslips. × 320. **b,** Positive immunoreaction with cytokeratin-8 showing a similar pattern of staining as that shown for the TPA antigen at 34th passage. Cell culture on coverslips. × 240. **c,** Positive immunolocalisation of CEA in 8701-BC cells at passage 38. Cells appear reactive for CEA although not all show the same stain intensity. Cell culture on coverslips. × 240.

**Figure 7 a,** Southern blotting analysis of DNA cut with EcoRI restriction enzyme: lane 1. PAF (human fibroblast cell line); lane 2, 8701-BC 45th passage; lane 3, DAUDI (human lymphoma cell line). **b,** Northern blotting analysis of cellular RNA hybridised to the c-*myc* probe: lane 1, PAF; lane 2, 8701-BC; lane 3, HL60 (human promyelocytic cell line); lane 4, DAUDI.

**Figure 8** Trypsin-Giemsa banding of 8701-BC cells. **A**, A metaphase spread in which the two marker chromosomes are indicated. Using this method of banding, only five normal chromosomes out of the 55 could be identified **B**, a no. 10(f), two no. 12s (g and h), an X chromosome (i) and a no. 19 (j). The chromosome (a) has a normal 1q region and possibly 2q material. (b) and (c) are both 1p regions, (d) is a no. 5 with a deletion of the short arms. Chromosome (e) is a no. 8 with additional material (possible 3q) on the long arms. The chromosomes in **C** were not identified.

line was achieved by adapting dissociative techniques to obtain significant improvements in cell cultivation. One advantage was the elimination of pronase and other general proteases which apparently impair cell viability, and the addition of a high concentration of FCS and TBP which facilitated cell growth. After a few passages (from the 6th on) the presumptive neoplastic cell population gave pure cultures as a result of their higher proliferative rate compared to the mesenchymal and normal epithelial cells.

The epithelial origin of 8701-BC cells was supported by their general morphological features, as well as ultrastructural characteristics; the ability to form colonies and 'spikes', the formation of interdigitated cell contacts and junctional complexes, as well as the production of inter- and intracellular canalicula lined by microvilli. Most of these structures are generally reported in tumour-derived cell cultures (see Engel & Yung, 1978), and have been maintained by 8701-BC beyond their 100th passage over almost 3 years.

Immunostaining for CEA, TPA and cytokeratin 8 antigenicity confirmed the epithelial nature and purity of 8701-BC cells. Despite reservations about CEA specificity as a tumour marker (Chretien, 1976) both CEA and TPA have been used singly (Bjorklund *et al.*, 1982) or combined (Luthgens & Schlegel, 1983; Oehr *et al.*, 1984) to characterise tumour cells. Goldberg *et al.* (1978) reported that positive CEA reactions in normal epithelial cells were artifactual and Kuhajda *et al.* (1983) also reported that normal breast cells were negative for CEA staining. These results are in accord with our observations since neoplastic cells gave positive reactions for CEA both in histological tumour sections (data not shown) and in cultures of 8701-BC, whereas normal human breast epithelial cells were negative. Positive cytokeratin 8 and TPA reactions had the same cellular distribution as expected from their reported homology (Luning & Nilsson, 1983). In contrast, the negative HLA reaction for 8701-BC cells was not unexpected since this was reported in neoplastic tissue of 45 patients with thyroid carcinoma (Larsen *et al.*, 1986) and alterations in HLA expression are a recognised property of many human tumours (Ruiz-Cabello *et al.*, 1988).

In addition to the morphological characterization of these cells *in vitro* the chromosome studies have demonstrated an abnormal chromosome number of between 55 and 60 with numerous chromosomal rearrangements for the 8701-BC cells. The recently described VHB-1 cell line was also reported to have an abnormal karyotype (Vandewalle *et al.*, 1987), but with numerical differences to the karyotype reported here. The proliferative properties of the 8701-BC falls into the range expected for tumour cell populations, and is similar to that of the VHB-1 line. Although it was reported that one in five human breast carcinoma cell lines had an amplified c-*myc* gene (Kozbor & Croce, 1984) we found no particular involvement of this oncogene in 8701-BC as judged by its apparent lack of amplification in relation to other human cell lines.

Our characterisation of the 8701-BC cell line to date, especially its morphological similarities with breast carcinoma cells *in situ*, its abnormal karyotype, and its continued rate of proliferation after 3 years and more than 100 passages *in vitro*, suggest the establishment of a breast carcinoma cell line. Moreover, the ability of this tumour cell line to produce homotrimer collagen *in vitro* (Minafra *et al.*, 1988) and the ability of this matrix component to modify the morphological and behavioural properties of 8701-BC cells *in vitro* (Schillaci *et al.*, 1989) suggests that this tumour cell line would offer many advantages for the study of neoplastic cell:stromal matrix interactions. Indeed, the production of an embryonic-type collagen may well relate to an oncofetal-type transformation which may be associated with invasive or metastatic properties. The tumorigenic behaviour of 8701-BC in nude mice would facilitate such studies and these experiments are now in progress.

Financial support for this work by Associazione italiana per la Ricerca sul Cancro (AIRC) and by the Administrazione Provinciale di Palermo is gratefully acknowledged. The authors wish to express their gratitude to Professor A. Chiarini (University of Palermo) for discussion and assistance in overcoming culturing problems, and for hospitality in his laboratory.

### References

BERGER, S.L. & BIRKENMEIR, C.S. (1979). Inhibition of intractable nucleases with ribonucleoside-vanadyl complexes: isolation of messenger ribonucleic acid from resting lymphocytes. *Biochemistry*, **18**, 5143.

BJORKLUND, V., BJORKLUND, B., WITTEKIND, Ch. & VON KLEIST, S. (1982). Immunohistochemical localization of tissue polypeptide antigen (TPA) and carcinoembryonic antigen (CEA) in breast cancer. *Acta Pathol. Scand.*, **A6**, 471.

BLOOM, H.J.G. & RICHARDSON, W.W. (1957). Histological grading and prognosis in breast cancer. *Br. J. Cancer*, **11**, 359.

COLLINS, S., GALLO, R. & GALLAGER, R. (1977). Continuous growth and differentiation of human myeloid leukaemic cells in suspension culture. *Nature*, **270**, 347.

CHREITEN, P.B. (1976) *Centennial Conference on Laryngeal Cancer*, Appleton: New York.

DALLA FAVERA, R., MARTINOTTI, S., GALLO, R., ERIKSON, J. & CROCE, C.M. (1983). Translocation and rearrangements of c-myc oncogene locus in human undifferentiated B-cell lymphomas. *Science*, **219**, 963.

DOBRYNIN, Y.V. (1963). Establishment and characteristic of cell strains from some epithelial tumours of human origin. *J. Natl Cancer Inst.*, **31**, 1173.

EASTY, G.C., EASTY, D.M., MONOGAM, P., ORMEROD, M.G. & NEVILLE, A.M. (1980). Preparation and identification of human epithelial cells in culture. *Int. J. Cancer*, **26**, 577.

ENGEL, L.W. & YUNG, N.A. (1978). Human breast carcinoma cells in continuous culture: a review. *Cancer Res.*, **38**, 4327.

FEO, S., HARVEY, R., SHOWE, L. & CROCE, C.M. (1986). Regulation of translocated c-*myc* genes transfected into plasmacytoma cells. *Proc. Natl Acad. Sci. USA*, **83**, 706.

GOLDBERG, D.M., SHARKEY, R.M. & PRIMUS, F.J. (1978). Immunocytochemical detection of carcinoembryonic antigen in conventional histopathology specimens. *Cancer*, **42**, 1546.

HACKETT, A.J., SMITH, H.S., SPRINGES, E.L. and 4 others (1977). Two syngeneic cell lines from human breast tissue; the aneuploid mammary epithelial (HS578T) and the diploid myoepithelial (Hs57sBst) cell lines. *J. Natl Cancer Inst.*, **58**, 1795.

HSU, S.M., RAINE, L. & FANGER, H. (1981). The use of avidin/biotin peroxidase complex (ABC) in immunoperoxidase techniques: a comparison between ABC and unlabelled antibody (PAP) procedures. *J. Histochem. Cytochem.*, **29**, 577.

KOZBOR, D. & CROCE, C.M. (1984). Amplification of the c-*myc* oncogene in one of five human breast carcinoma cell lines. *Cancer Res.*, **44**, 438.

KUHAJDA, F.P., OFFUTT, L.E. & MENDELSOHN, G. (1983). The distribution of carcinoembryonic antigen in breast carcinoma. Diagnostic and prognostic implications. *Cancer*, **52**, 21257.

LARSEN, B., THOMPSON, C., HWAN, A. & FARID, N.R. (1986). Lack of association of HLA with thyroid cancer. An effect of iodine sufficiency and safe environment? *Tissue Antigens*, **28**, 298.

LASFARGUES, E.W., CONTINKO, W.G. & REDFIELD, E.S. (1978). Isolation of two human tumor epithelial cell lines from solid breast carcinoma. *J. Natl Cancer Inst.*, **61**, 967.

LUNING, B. & NILSSON, U. (1983). Sequence homology between tissue polypeptide antigen (TPA) and intermediate filament (IF) proteins. *Acta Chem. Scand.*, **B37**, 731.

LUTHGENS, M. & SCHLEGEL, G. (1983). Sensitivity and specificity of TPA, CEA and the index of both markers in various malignancies. In *Protides*, Peeters, H. (ed), p. 475. Pergamon Press: Oxford.

MINAFRA, S., PUCCI-MINAFRA, I., SCIARRINO, S., MORELLO, V. & TOMASINO, R.M. (1984*a*). Analisi morofunzionale e biochimica dello stroma del carcinoma duttale infiltrante della mammella umana. *Riv. Anat. Patol. Oncol.*, **43**, 341.

MINAFRA, S., PUCCI-MINAFRA, I., TOMASINO, R.M. & SCIARRINO, S. (1984*b*). Collagen composition in the ductal infiltrating carcinoma of the human breast. *Cell Biol. Intern. Rep.*, **8**, 79.

MINAFRA, S., LUPARELLO, C., RALLO, F. & PUCCI-MINAFRA, I. (1988). Collagen biosynthesis by a breast carcinoma cell strain and biopsy fragments of the primary tumour. *Cell Biol. Intern. Rep.*, **12**, 895.

MOLL, R., FRANKE, W.W., SCHILLER, D.L., GEIGER, B. & KREPLER, R. (1982). The catalog of human cytokeratins: patterns of expression in normal epithelia, tumors and cultured cells. *Cell*, **31**, 11.

MONAGHAN, P., WHITEHEAD, R.H., PERUSINGHE, N. & O'HARE, M.J. (1985). An immunocytochemical and ultrastructural study of heterogeneity in the human breast carcinoma cell line PMC42. *Cancer Res.*, **45**, 5088.

NORDQUIST, R.E., ISHMAEL, D.R. & LOVIG, G.A. (1975). The tissue culture and morphology of human breast tumor cell line BOT-2. *Cancer Res.*, **35**, 3100.

OEHR, P., BONNEN, U. & WINKLER, C. (1984). Prognostic values of TPA, CEA and their combination by product formation in different cancer location. In *Protides*, Peeters, H. (ed) p. 755. Pergamon Press: Oxford.

PUCCI-MINAFRA, I., LUPARELLO, C., SCIARRINO, S., TOMASINO, R.M. & MINAFRA, S. (1985). Quantitative determination of collagen types present in the ductal infiltrating carcinoma of human mammary gland. *Cell Biol. Intern. Rep.*, **9**, 291.

PUCCI-MINAFRA, I., MINAFRA, S., TOMASINO, R.M., SCIARRINO, S. & TINERVIA, R. (1986). Collagen changes in the ductal infiltrating (scirrhous) carcinoma of the human breast. A possible role played by type I-trimer collagen on the invasive growth. *J. Submicrosc. Cytol.*, **18**, 795.

PUCCI-MINAFRA, I., LUPARELLO, C., SCHILLACI, R. & SCIARRINO, S. (1987). Ultrastructural evidence of collagenolytic activity in ductal infiltrating carcinoma of the human breast. *Int. J. Cancer.*, **39**, 599.

RUDLAND, P.S., HALLOWES, R.C., COX. S.A., ORMEROD, E.J. & WARBURTON, M.J. (1985). Loss of production of myoepithelial cells and basement membrane proteins but retention of response to certain growth factors and hormones by a new malignant human breast cancer cell strain. *Cancer Res.*, **45**, 3864.

RUIZ-CABELLO, F., LOPEZ NEVOT, M.A. & GARRIDO, F. (1988). MHC Class I and II gene expression on human tumours. *Cancer Metastasis Adv. Exp. Med.*, **233**, 119.

SCHILLACI, R., LUPARELLO, C. & MINAFRA, S. (1989). Type I and I-trimer collagens as substrates for breast carcinoma cells in culture. *Eur. J. Cell Biol.*, **48**, 135.

SOULE, H.D., VASQUEZ, J., LONG, A., ALBERT, S. & BRENNAN, M.J. (1973). A human cell line from a pleural effusion derived from a breast carcinoma. *J. Natl Cancer Inst.*, **51**, 1409.

SOUTHERN, E.M. (1975). Detection of specific sequences among DNA fragments separated by gel electrophoresis. *J. Molec. Biol.*, **98**, 503.

STERNBERGER, L.S.A. (1979). *Immunocytochemistry*, 2nd edn. Wiley: New York.

TREMPE, G. & FOGH, J. (1973). Variation of characteristic of human tumor cell lines derived from similar tumors. *In Vitro*, **8**, 433.

VANDEWALLE, B., COLLYN D'HOUGHE, M., SAVARY, J.B. and 5 others (1987). Establishment and characterization of a new cell line (VHB-1) derived from a primary breast carcinoma. *J. Cancer Res. Clin. Oncol.*, **113**, 550.

WEBER, K., OSBORN, M., MOLL, R., WIKLUND, B. & LUNING, B. (1984). Tissue polypeptide antigen (TPA) is related to the non-epidermal keratina 8, 18 and 19 typical of simple and non-squamous epithelium. *EMBO J.*, **3**, 2707.


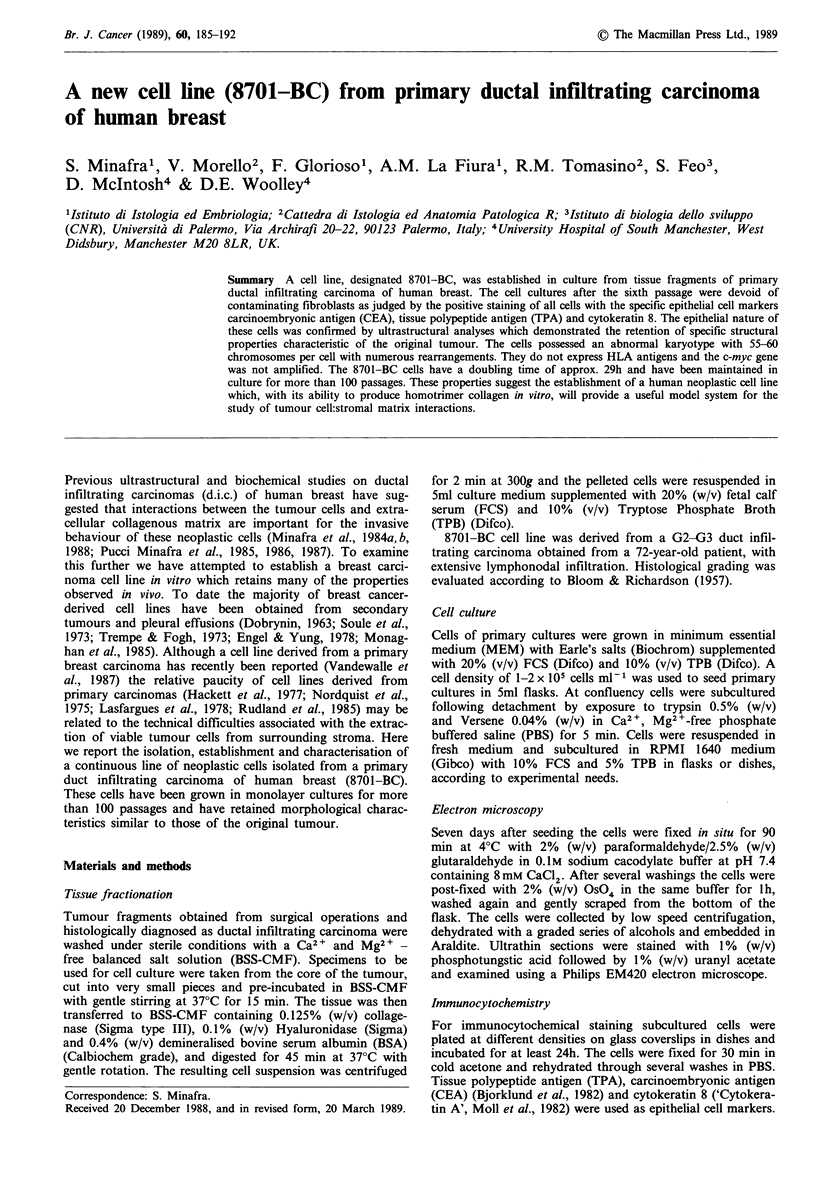

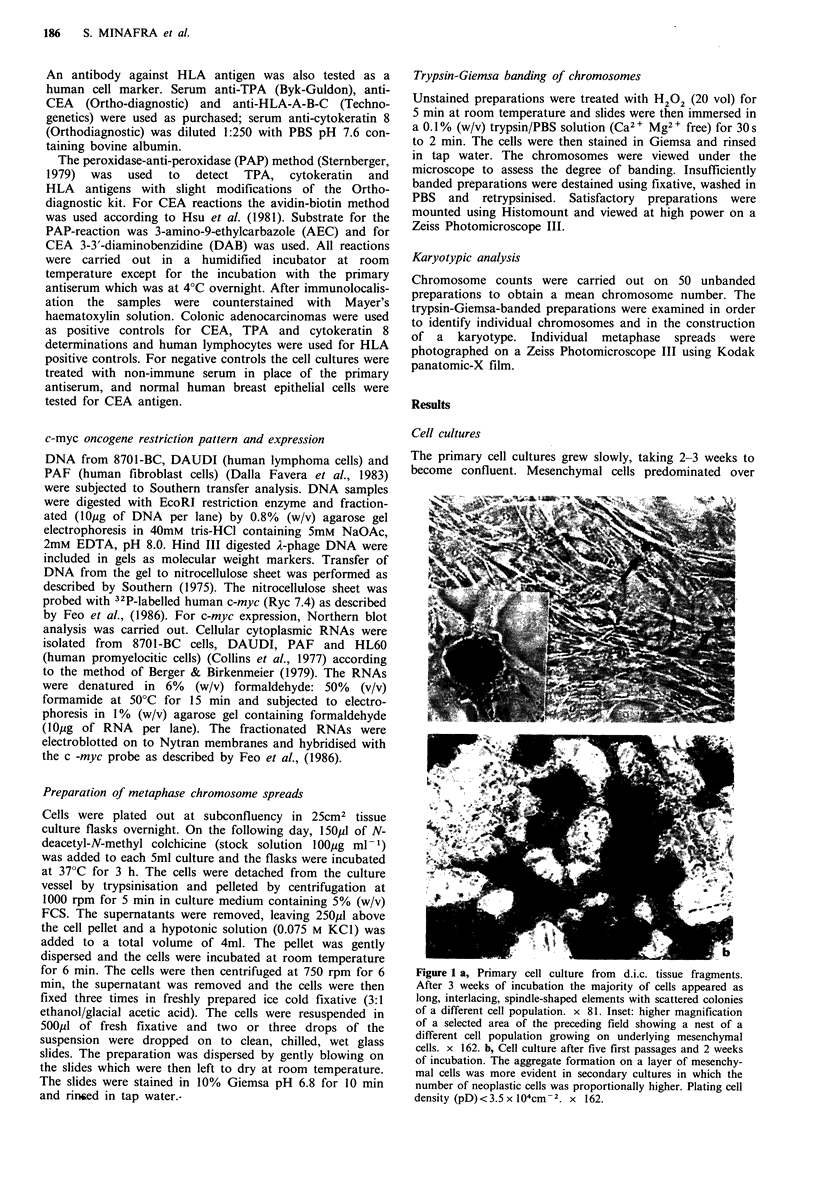

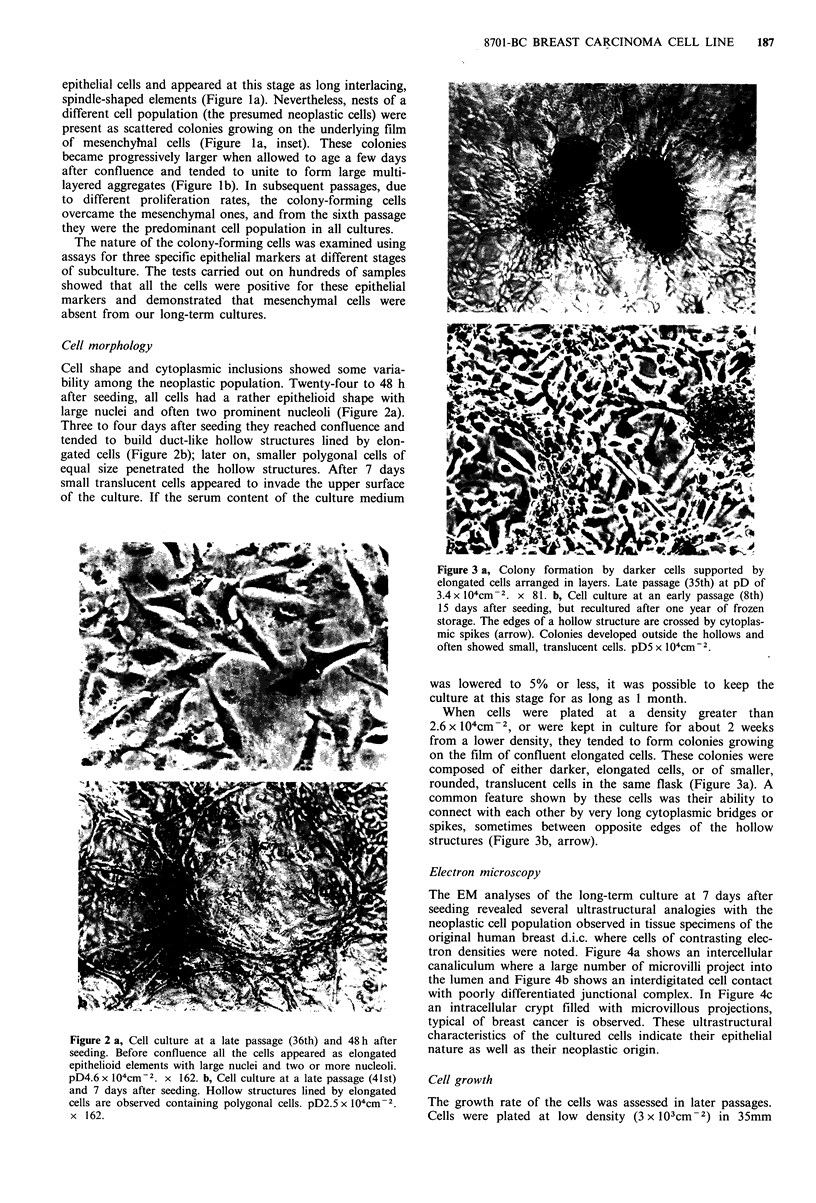

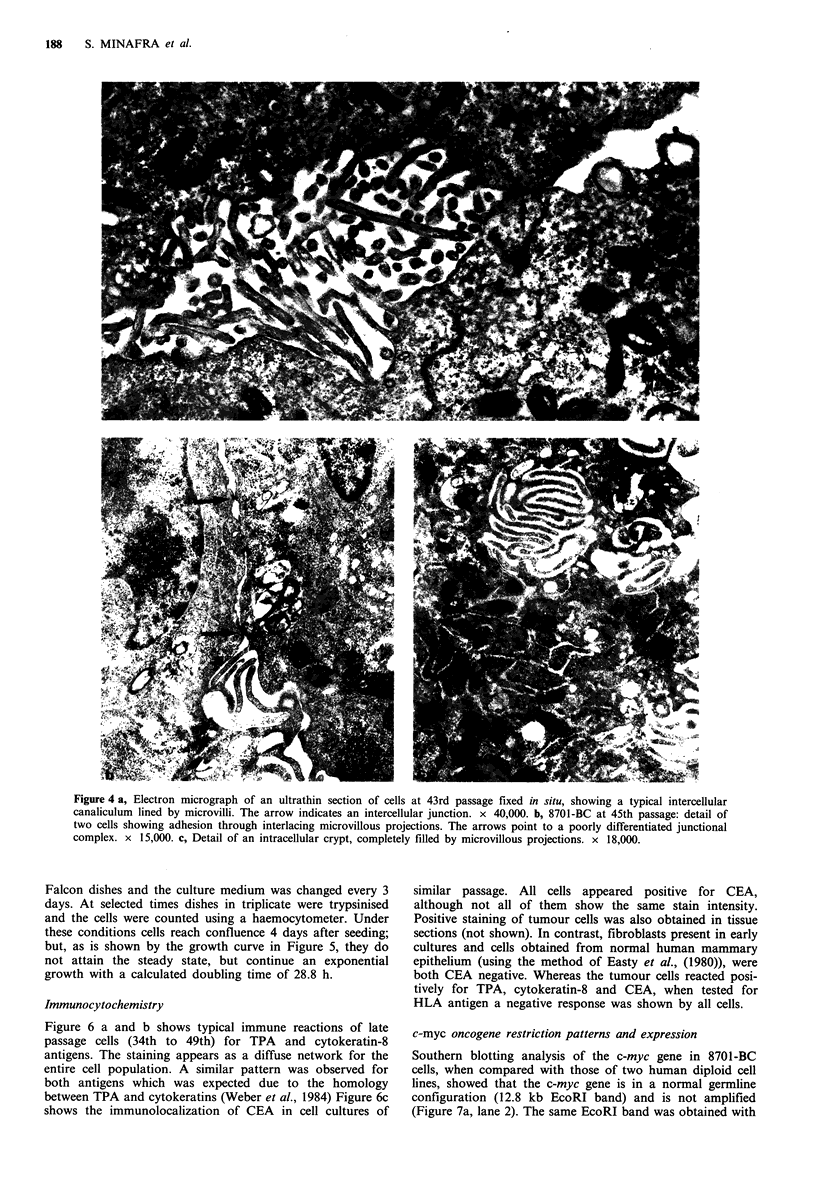

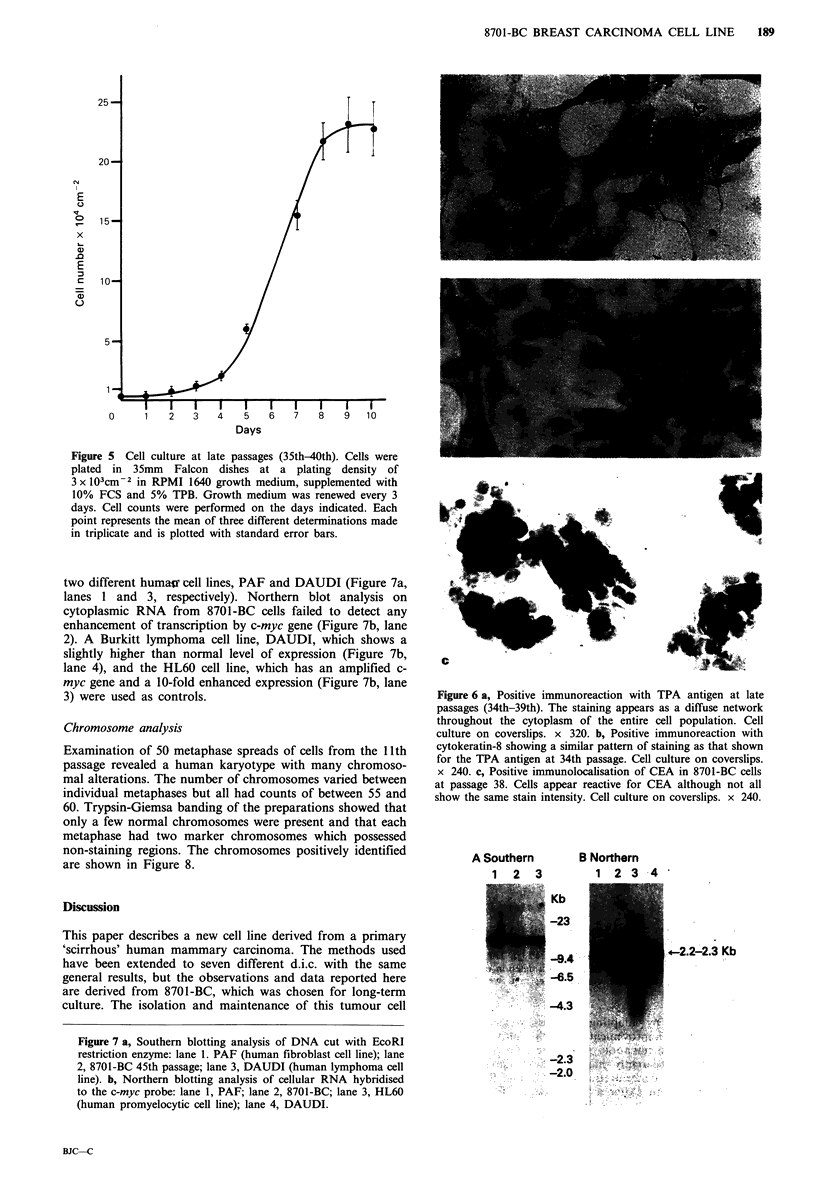

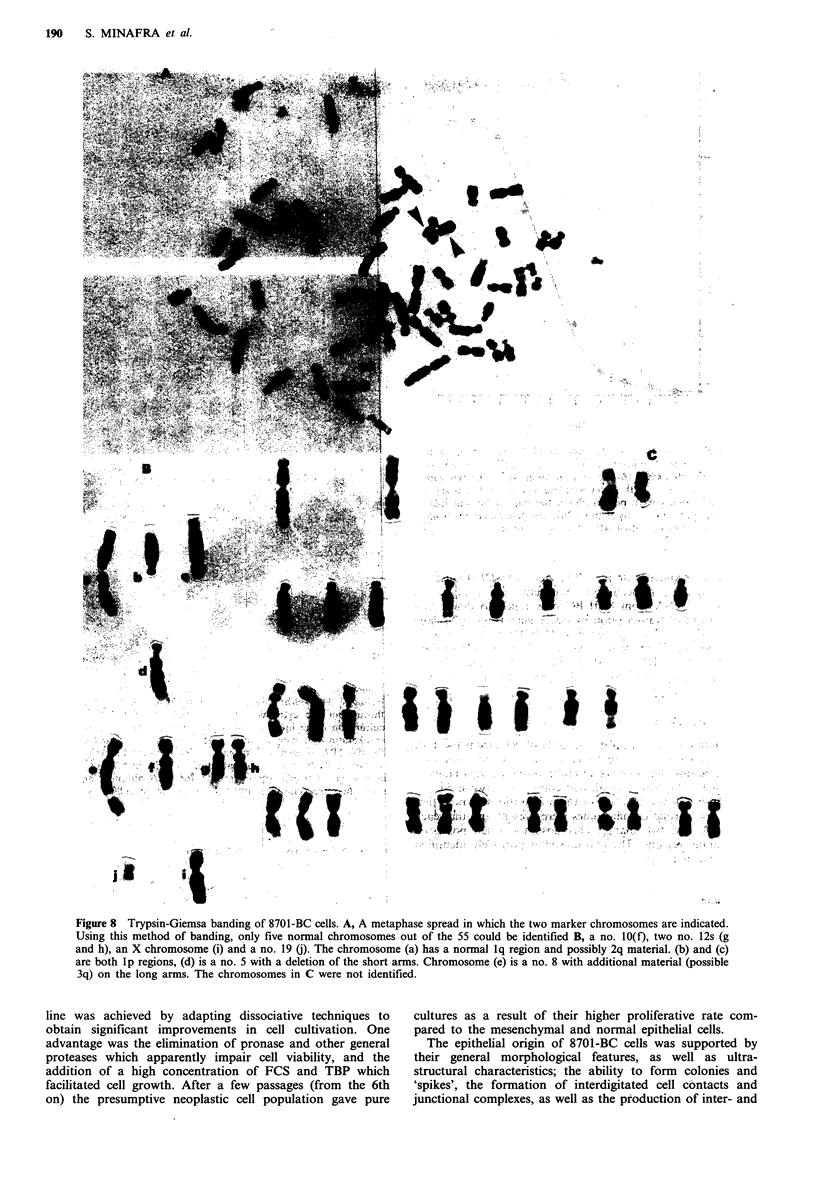

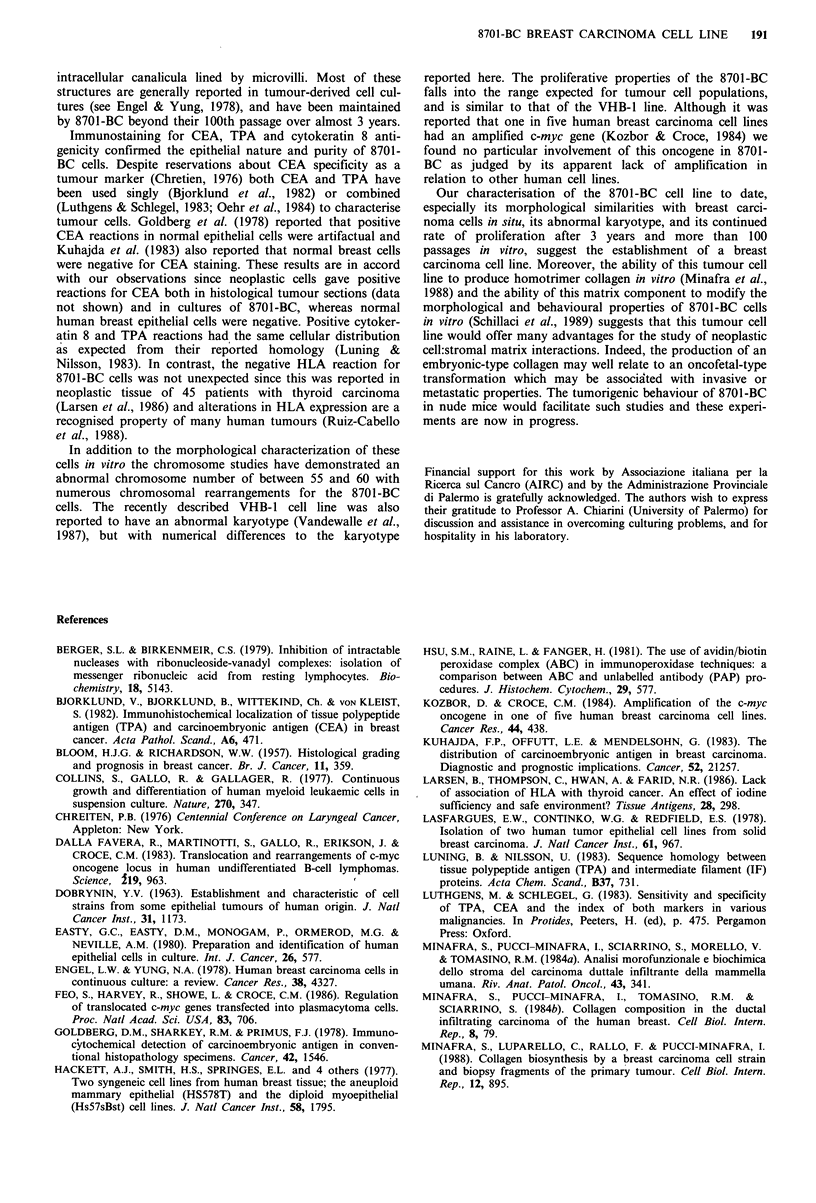

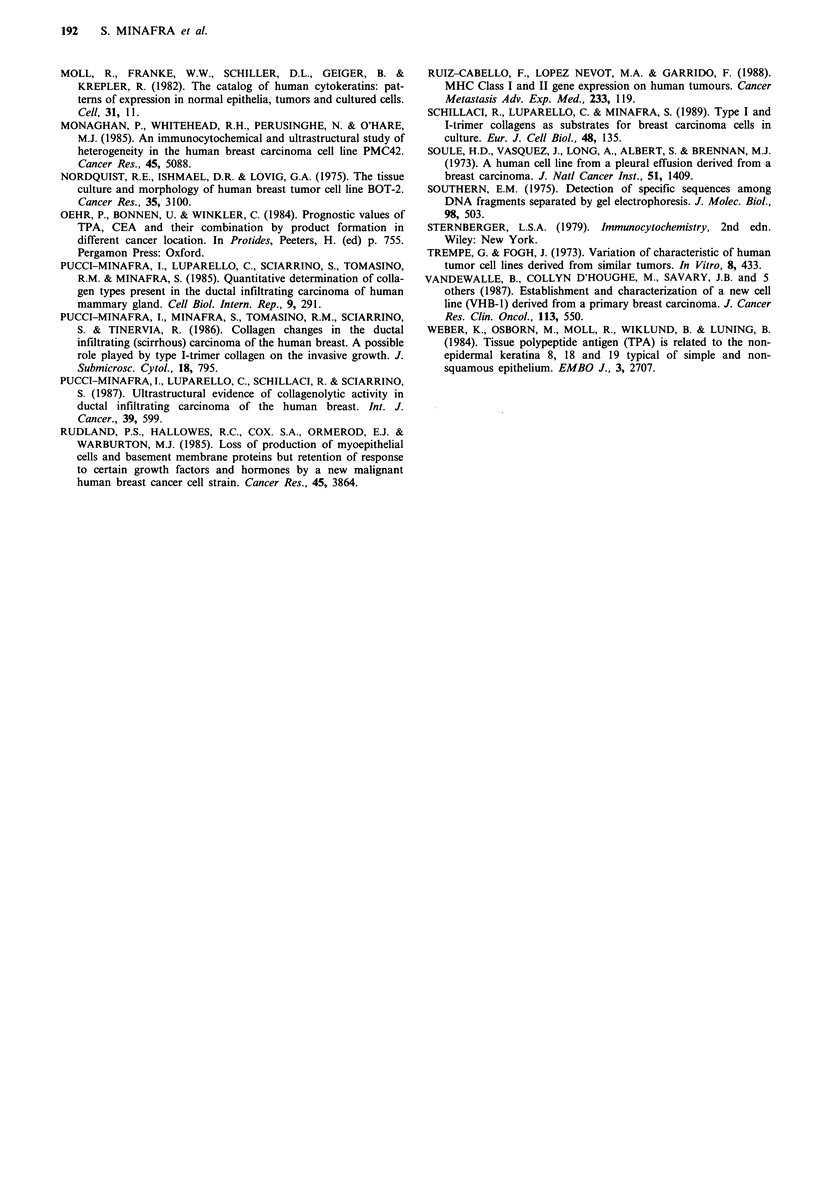


## References

[OCR_00644] BLOOM H. J., RICHARDSON W. W. (1957). Histological grading and prognosis in breast cancer; a study of 1409 cases of which 359 have been followed for 15 years.. Br J Cancer.

[OCR_00632] Berger S. L., Birkenmeier C. S. (1979). Inhibition of intractable nucleases with ribonucleoside--vanadyl complexes: isolation of messenger ribonucleic acid from resting lymphocytes.. Biochemistry.

[OCR_00638] Björklund V., Björklund B., Wittekind C., von Kleist S. (1982). Immuno-histochemical localization of tissue polypeptide antigen (TPA) and carcino-embryonic antigen (CEA) in breast cancer. A comparative study.. Acta Pathol Microbiol Immunol Scand A.

[OCR_00648] Collins S. J., Gallo R. C., Gallagher R. E. (1977). Continuous growth and differentiation of human myeloid leukaemic cells in suspension culture.. Nature.

[OCR_00663] DOBRYNIN Y. V. (1963). ESTABLISHMENT AND CHARACTERISTICS OF CELL STRAINS FROM SOME EPITHELIAL TUMORS OF HUMAN ORIGIN.. J Natl Cancer Inst.

[OCR_00659] Dalla-Favera R., Martinotti S., Gallo R. C., Erikson J., Croce C. M. (1983). Translocation and rearrangements of the c-myc oncogene locus in human undifferentiated B-cell lymphomas.. Science.

[OCR_00668] Easty G. C., Easty D. M., Monaghan P., Ormerod M. G., Neville A. M. (1980). Preparation and identification of human breast epithelial cells in culture.. Int J Cancer.

[OCR_00673] Engel L. W., Young N. A. (1978). Human breast carcinoma cells in continuous culture: a review.. Cancer Res.

[OCR_00677] Feo S., Harvey R., Showe L., Croce C. M. (1986). Regulation of translocated c-myc genes transfected into plasmacytoma cells.. Proc Natl Acad Sci U S A.

[OCR_00682] Goldenberg D. M., Sharkey R. M., Primus F. J. (1978). Immunocytochemical detection of carcinoembryonic antigen in conventional histopathology specimens.. Cancer.

[OCR_00687] Hackett A. J., Smith H. S., Springer E. L., Owens R. B., Nelson-Rees W. A., Riggs J. L., Gardner M. B. (1977). Two syngeneic cell lines from human breast tissue: the aneuploid mammary epithelial (Hs578T) and the diploid myoepithelial (Hs578Bst) cell lines.. J Natl Cancer Inst.

[OCR_00693] Hsu S. M., Raine L., Fanger H. (1981). Use of avidin-biotin-peroxidase complex (ABC) in immunoperoxidase techniques: a comparison between ABC and unlabeled antibody (PAP) procedures.. J Histochem Cytochem.

[OCR_00699] Kozbor D., Croce C. M. (1984). Amplification of the c-myc oncogene in one of five human breast carcinoma cell lines.. Cancer Res.

[OCR_00709] Larsen B., Thompson C., Kwan A., Farid N. R. (1986). Lack of association of HLA with thyroid cancer. An effect of iodine sufficiency and safe environment?. Tissue Antigens.

[OCR_00714] Lasfargues E. Y., Coutinho W. G., Redfield E. S. (1978). Isolation of two human tumor epithelial cell lines from solid breast carcinomas.. J Natl Cancer Inst.

[OCR_00719] Lüning B., Nilsson U. (1983). Sequence homology between tissue polypeptide antigen (TPA) and intermediate filament (IF) proteins.. Acta Chem Scand B.

[OCR_00742] Minafra S., Luparello C., Rallo F., Pucci-Minafra I. (1988). Collagen biosynthesis by a breast carcinoma cell strain and biopsy fragments of the primary tumour.. Cell Biol Int Rep.

[OCR_00750] Moll R., Franke W. W., Schiller D. L., Geiger B., Krepler R. (1982). The catalog of human cytokeratins: patterns of expression in normal epithelia, tumors and cultured cells.. Cell.

[OCR_00756] Monaghan P., Whitehead R. H., Perusinghe N., O'Hare M. J. (1985). An immunocytochemical and ultrastructural study of heterogeneity in the human breast carcinoma cell line PMC42.. Cancer Res.

[OCR_00762] Nordquist R. E., Ishmael D. R., Lovig C. A., Hyder D. M., Hoge A. F. (1975). The tissue culture and morphology of human breast tumor cell line BOT-2.. Cancer Res.

[OCR_00773] Pucci Minafra I., Luparello C., Sciarrino S., Tomasino R. M., Minafra S. (1985). Quantitative determination of collagen types present in the ductal infiltrating carcinoma of human mammary gland.. Cell Biol Int Rep.

[OCR_00779] Pucci Minafra I., Minafra S., Tomasino R. M., Sciarrino S., Tinervia R. (1986). Collagen changes in the ductal infiltrating (scirrhous) carcinoma of the human breast. A possible role played by type I trimer collagen on the invasive growth.. J Submicrosc Cytol.

[OCR_00786] Pucci-Minafra I., Luparello C., Schillaci R., Sciarrino S. (1987). Ultrastructural evidence of collagenolytic activity in ductal infiltrating carcinoma of the human breast.. Int J Cancer.

[OCR_00794] Rudland P. S., Hallowes R. C., Cox S. A., Ormerod E. J., Warburton M. J. (1985). Loss of production of myoepithelial cells and basement membrane proteins but retention of response to certain growth factors and hormones by a new malignant human breast cancer cell strain.. Cancer Res.

[OCR_00799] Ruiz-Cabello F., Nevot M. A., Garrido F. (1988). MHC class I and II gene expression on human tumors.. Adv Exp Med Biol.

[OCR_00804] Schillaci R., Luparello C., Minafra S. (1989). Type I and I-trimer collagens as substrates for breast carcinoma cells in culture. Effect on growth rate, morphological appearance and actin organization.. Eur J Cell Biol.

[OCR_00809] Soule H. D., Vazguez J., Long A., Albert S., Brennan M. (1973). A human cell line from a pleural effusion derived from a breast carcinoma.. J Natl Cancer Inst.

[OCR_00814] Southern E. M. (1975). Detection of specific sequences among DNA fragments separated by gel electrophoresis.. J Mol Biol.

[OCR_00827] Vandewalle B., Collyn d'Hooghe M., Savary J. B., Vilain M. O., Peyrat J. P., Deminatti M., Delobelle-Deroide A., Lefebvre J. (1987). Establishment and characterization of a new cell line (VHB-1) derived from a primary breast carcinoma.. J Cancer Res Clin Oncol.

[OCR_00833] Weber K., Osborn M., Moll R., Wiklund B., Lüning B. (1984). Tissue polypeptide antigen (TPA) is related to the non-epidermal keratins 8, 18 and 19 typical of simple and non-squamous epithelia: re-evaluation of a human tumor marker.. EMBO J.

